# miRNA-based signatures in cerebrospinal fluid as potential diagnostic tools for early stage Parkinson’s disease

**DOI:** 10.18632/oncotarget.24736

**Published:** 2018-04-03

**Authors:** Marcia Cristina T. dos Santos, Miguel Arturo Barreto-Sanz, Bruna Renata S. Correia, Rosie Bell, Catherine Widnall, Luis Tosar Perez, Caroline Berteau, Claudia Schulte, Dieter Scheller, Daniela Berg, Walter Maetzler, Pedro A.F. Galante, Andre Nogueira da Costa

**Affiliations:** ^1^ Experimental Medicine and Diagnostics, Global Exploratory Development, UCB Biopharma SPRL, Braine-l'Alleud, Belgium; ^2^ SimplicityBio SA, Monthey, Switzerland; ^3^ Hospital Sirio Libanes, São Paulo, Brazil; ^4^ Centre for Misfolding Diseases, University of Cambridge, Cambridge, UK; ^5^ Leeds Institute of Biomedical and Clinical Sciences, University of Leeds, Leeds, UK; ^6^ Bioanalytical Sciences, Non Clinical Development, UCB Biopharma SPRL, Belgium; ^7^ Hertie Institute for Clinical Brain Research, Department of Neurodegeneration, University of Tuebingen and German Center for Neurodegenerative Diseases, Tuebingen, Germany; ^8^ Consultancy Neuropharm, Neukirchener, Neuss, Germany; ^9^ Department of Neurology, Christian-Albrechts-University Kiel, Kiel, Germany

**Keywords:** exosomal miRNA, early stage PD diagnosis, CSF, machine learning

## Abstract

Parkinson’s Disease is the second most common neurodegenerative disorder, affecting 1–2% of the elderly population. Its diagnosis is still based on the identification of motor symptoms when a considerable number of dopaminergic neurons are already lost. The development of translatable biomarkers for accurate diagnosis at the earliest stages of PD is of extreme interest. Several microRNAs have been associated with PD pathophysiology. Consequently, microRNAs are emerging as potential biomarkers, especially due to their presence in Cerebrospinal Fluid and peripheral circulation. This study employed small RNA sequencing, protein binding ligand assays and machine learning in a cross-sectional cohort comprising 40 early stage PD patients and 40 well-matched controls. We identified a panel comprising 5 microRNAs (Let-7f-5p, miR-27a-3p, miR-125a-5p, miR-151a-3p and miR-423-5p), with 90% sensitivity, 80% specificity and 82% area under the curve (AUC) for the differentiation of the cohorts. Moreover, we combined miRNA profiles with hallmark-proteins of PD and identified a panel (miR-10b-5p, miR-22-3p, miR-151a-3p and α-synuclein) reaching 97% sensitivity, 90% specificity and 96% AUC. We performed a gene ontology analysis for the genes targeted by the microRNAs present in each panel and showed the likely association of the models with pathways involved in PD pathogenesis.

## INTRODUCTION

Parkinson’s disease (PD) is the second most common neurodegenerative disease affecting over 5 million people worldwide [1]. The disease is characterized by progressive death of dopaminergic neurons and the presence of intra-cytoplasmic inclusions consisting mostly of α-synuclein (α-syn) within many areas of the brain, including the substantia nigra (SN) [2–4]. PD is considered a complex and heterogeneous neurodegenerative disease that results in impairments in movement and cognitive capability [5]. Currently, PD diagnosis is primarily based on the presence of two of the three major motor symptoms: bradykinesia, rigidity and tremor at rest [6]. Nevertheless, scales employed in clinical diagnosis are subjective and can only be detected when motor features are present and 60–90% of dopaminergic neurons are already lost [7, 8]. The discovery of a reliable quantitative diagnostic test for PD is of extreme interest. Molecular biomarkers that are objective and measurable can be potential clinical tools to support PD clinical diagnosis, especially during the earliest stages of the disease.

Cerebrospinal Fluid (CSF) represents an optimal source of biomarkers of neurodegenerative diseases and has been extensively employed in PD biomarker research [1]. However, investigations are heavily based on proteins related to PD pathogenesis. For instance, studies have highlighted altered levels of α-syn and DJ-1 in PD patients compared with controls [9–14]. Furthermore, due to assay incompatibility and lack of standardized protocols, results are conflicting and do not show robustness for use as clinical biomarkers [15].

An emerging area of research is investigating microRNAs (miRNAs) as possible biomarkers of PD. miRNAs are small 21–24 nucleotide non-coding RNAs that regulate gene expression by inhibiting translation of target genes [16]. A large number of miRNAs are brain specific and have also been found in various biofluids [17, 18]. Altered expression of miRNAs in the brain has been described in several neurological disorders and neurodegenerative diseases [19, 20]. Due to their ability to cross the Blood Brain Barrier (BBB) and being both free in circulation as well as present in exosomes, miRNAs have the potential to be valuable biomarkers providing insights to the pathological signs detected in the Central Nervous System (CNS) [21]. miRNAs have been used as biomarkers for a potential non-invasive diagnosis of several disorders [22–25], but only a limited number of miRNAs have been implicated with PD [15].

In this study, we aimed at developing an algorithm based on molecular profiles which could improve the diagnosis of early stage PD. To this end, we analyzed the CSF miRNA and protein profiles, by means of small RNA-sequencing and ligand binding assays, respectively, of a cross-sectional cohort composed of early stage PD patients (*n = 40*) and matched control subjects (*n = 40*). Through the combination of molecular and clinical endpoints and subsequent machine learning, we identified a panel of miRNAs with 90% diagnostic sensitivity, 80% diagnostic specificity and 82% ROC-AUC (Receiver Operating Characteristic-Area under the curve). Additionally, when combining one of the protein hallmarks of PD, α-syn, with miRNAs, we identified a subsequent panel with improved diagnostic accuracy with 97% diagnostic sensitivity, 90% diagnostic specificity and 96% ROC-AUC. Our panels showed strong robustness through scientific rationale and have great potential as clinical diagnostic biomarkers. Notably, through computational biology analysis, we show that these panels are associated with pathways, such as prion diseases and ubiquitin mediated proteolysis, proposed as key mechanistic regulators of PD pathology [26–31].

## RESULTS

### Variance analysis of the clinical information

The demographic characteristics of the 40 early stage PD patients and 40 control samples included in this study are summarized in Table 1. Among PD patients, the group consisted of 20 males and 20 females, ranging from 39 to 80 years in age with an average of 61 ± 1 years, H&Y median: 2 and UPDRS III median: 21. Similarly, the control group consisted of 20 males and 20 females, ranging from 42 to 83 years in age with an average of 64 ± 1 years. Analysis of variance revealed no significant source of variation in the expression data due to age, gender and disease duration.

**Table 1:** Cohort summary

	PD	Controls	*p* value
Individuals (*n*)	40	40	NA
gender (male in % (m/f))	50% (20/20)	50% (20/20)	NA
age (in years mean +/- SD)	61 ± 1	64 ± 1	0.0998
Disease duration (in years mean)	1.8 ± 1	NA	NA
H&Y (median)	2	NA	NA
UPDRS III (median)	21	NA	NA
α-syn (pg/mL)	506.1 ± 28^*^	868.4 ± 48^*^	< 0.0001
DJ-1 (pg/mL)	4988 ± 499^*^	7501 ± 619^*^	0.0023
UCHL1 (ng/mL)	0.71 ± 0.45^*^	0.66 ± 0.42^*^	0.209

*p* values are calculated with Pearson Chi Square or *t*-test.

^*^Mean ± SD.

A total of 80 individuals were include in this study, 40 early stage PD patients and 40 controls. Gender, age and disease duration were calculated for both groups and are presented below. Protein markers levels are represented by mean values plus SD (Standard Deviation). H&Y = Hoehn and Yahr staging and UPDRS III = Unified Parkinson’s Disease Rating Scale.

### miRNA expression profile

We employed Next Generation Sequencing (NGS) to globally profile miRNAs in the CSF of early stage PD patients and controls. Given the low RNA content in the CSF samples, we customized the small RNA sequencing workflow to achieve successful sequencing runs starting from low input RNA. On average, 27 million reads were generated per sample. Read length distribution and annotation were evaluated per sample to ensure enrichment of miRNAs in the 20–24nt read fraction. We were able to detect the expression of a total 1683 miRNAs. From those, 389 passed the first exclusion criteria, which excluded all miRNAs with less than 5 read counts per sample. Of those, 301 miRNAs were expressed in all samples. We searched for miRNAs exclusively expressed by either controls or early stage PD, and none of the miRNAs were expressed in one group only. Additionally, we removed miRNAs that had the same expression patterns across groups, finalizing with a total of 121 miRNAs comprised in the final dataset used for analysis.

### Identification of a miRNA-based biomarker panel for the early diagnosis of PD

After processing and stringently filtered the miRNAs data, we used machine learning to identify a miRNA-based panel that could accurately distinguish early stage PD patients from controls.

**Figure 1:**
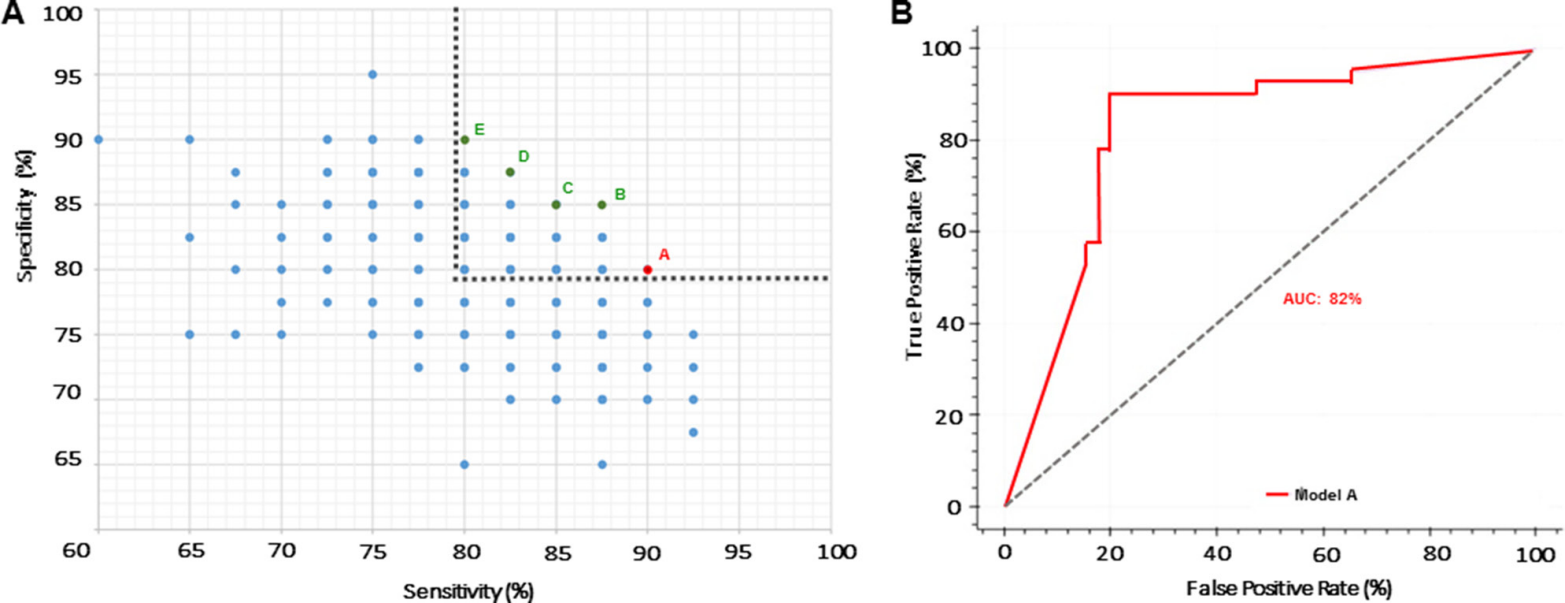
Selection of a robust miRNA-based panel (**A**) Pareto Efficiency highlighting miRNA-based models. Gray dashed line represents threshold used to select models with over 80% sensitivity and specificity. Red dot represents the selected model (Model A) with 90% sensitivity and 80% specificity. Green dots represent Models B-E. (**B**) ROC curve of selected Model A with AUC of 82%.

Through the combination of miRNAs and clinical endpoints, a total of 3200 models were created, trained and tested using Fuzzy Modeling, also known as fuzzy inference systems [32]. For the selection of the best models, we applied Fuzzy CoCo modeling to filter and exclude models with high complexity and difficult interpretation (such as models with large number of variables, complex relationships between the variables and subsequent interpretable interaction) [33]. The models fitting the defined criteria were subsequently subjected to a feature selection step, which revealed the 15 miRNAs most frequently found among all models (Table 2). Based on the top-ranking variables, 329 new models were created, trained and tested. In order to improve robustness, we applied advanced filtering pathways and focused on the models with diagnostic sensitivity and specificity values above 80%. This approach led to the identification of 5 preferential panels based on their robustness and complexity (Supplementary Figure 1A and Supplementary Table 1). From those, we selected Model A based on sensitivity, complexity and variable composition Figure 1A. Interestingly, Model A contains the 5 best ranking variables (Let-7f-5p, miR-125a-5p, miR-151a-3p, miR-27a-3p and miR-423-5p) and showed high predictive value with 90% diagnostic sensitivity, 80% diagnostic specificity and 82 and 89% positive and negative predicted values respectively (Supplementary Table 1). Receiver Operating Characteristic (ROC) curve analysis was performed to determine the diagnostic accuracy of the panels, which presented 82% of area under the curve (AUC) (Figure 1B).

**Table 2:** Top ranking variables

miRNAs		miRNAs+α-syn	
Ranking	Variable	Ranking	Variable
1	Let-7f-5p	1	α-syn
2	miR-423-5p	2	miR-26b-5p
3	miR-27a-3p	3	miR-10b-5p
4	miR-151a-3p	4	miR-323a-3p
5	miR-125a-5p	5	miR-4654
6	miR-30c-5p	6	miR-203a-3p
7	miR-511-5p	7	miR-9-3p
8	miR-1911-5p	8	miR-152-3p
9	miR-382-5p	9	miR-423-3p
10	miR-335-5p	10	miR-95-3p
11	Let-7d-5p	11	miR-151a-3p
12	miR-101-3p	12	miR-182-5p
13	miR-4418	13	miR-1246
14	miR-95-3p	14	miR-22-3p
15	miR-10b-5p	15	miR-30e-3p

On the left, variables most frequent among models with miRNAs only. On the right, variables most frequent among models including miRNAs and α-syn.

### α-syn improves robustness of a miRNA-based panel

**Figure 2:**
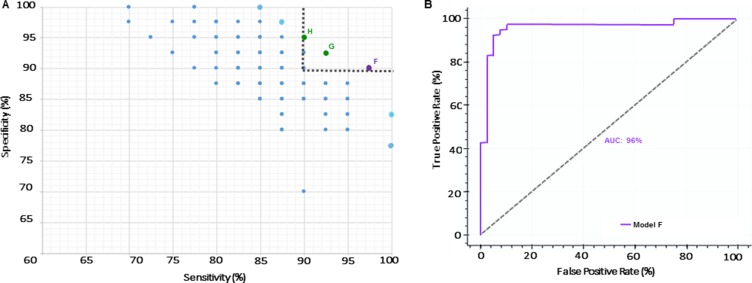
Inclusion of α-syn as a variable improves performance of a miRNA-panel (**A**) Pareto Efficiency of models including miRNAs and α-syn. Gray dashed line represents threshold used to select models with over 90% sensitivity and specificity. Purple dot represents the selected model (Model F) with 97% sensitivity and 90% specificity. Green dots represent Models G and H. (**B**) ROC curve of selected Model F with AUC of 96%.

We determined if the hallmark proteins of PD and neurodegeneration (DJ-1, UCHL1 and α-syn) could be combined with miRNA profiles to develop robust panels. We measured the protein levels in the CSF of all subjects (Table 1). We combined the proteins and performed a similar analysis as described above. A total of 1600 models were created, trained and tested. After applying Fuzzy CoCo modeling, we identified 335 models of which a feature selection step was applied and α-syn emerged as the most frequent variable among all models (Table 2). Subsequently, we focused our analysis on models with diagnostic sensitivity and specificity values above 90%. This approach led us to identify 3 models with high predictive values (Supplementary Figure 1B and Supplementary Table 2). From those, we selected Model F based on sensitivity, number of variables and complexity Figure 2A. Model F comprises miRNAs miR-10b-5p, miR-151a-3p, miR-22-3p and α-syn. The model presents 97% diagnostic sensitivity, 90% diagnostic specificity and 90 and 97% positive and negative predicted value respectively. ROC analysis revealed 96% AUC (Figure 2B).

### Pathways analyses of the miRNAs included in Model A and Model F

**Figure 3:**
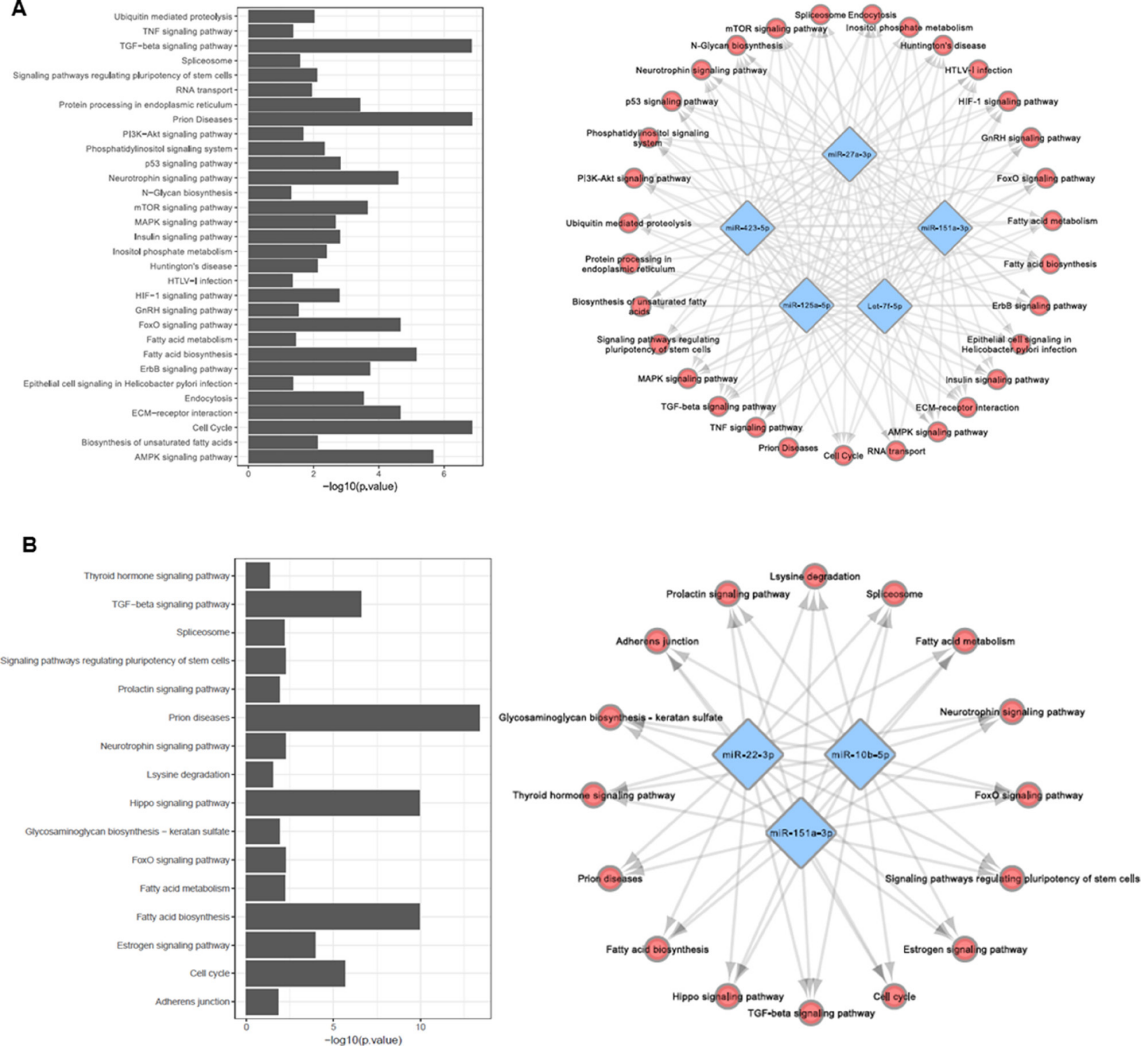
Selected models are targeting genes involved in several pathways associated with PD pathogenesis (**A**) Biological network representing pathways regulated by miRNAs present in Model A. (**B**) Biological network representing pathways regulated by miRNAs present in Model F. Bar charts represent the –log10(*p*-value) of enriched pathways.

We applied DIANA-TarBase [34] to identify all genes targeted by the miRNAs included in Model A and Model F. To this end, we considered only experimentally validated miRNA interactions. We identified 31 pathways involved in PD pathogenesis being regulated by the miRNAs proposed in model A (Figure 3A). Among the pathways regulated by the miRNAs presented in Model A, Prion disease (*p* < 0.001), TGF-beta signaling (*p* < 0.001) and cell cycle regulation (*p* < 0.001) were the most prominent. Ubiquitin mediated proteolysis (*p* < 0.01), Neurotrophin signaling (*p* < 0.01), mTOR signaling (*p* < 0.01), AMPK signaling (*p* < 0.01), FoxO signaling (*p* < 0.01) and Huntington’s Disease pathway (*p* < 0.01) were also enriched in our analysis.

For model F we identified 16 pathways being regulated by the miRNAs proposed in the model (Figure 3B). For Model F, Prion disease pathways (*p* < 0,001), Hippo signaling (*p* < 0,001) and Fatty acid biosynthesis (*p* < 0,001) were enriched in the pathways analysis.

## DISCUSSION

Currently, PD diagnosis still relies on the clinical diagnosis based on the emergence of motor symptoms; its accuracy is reported as not beyond 75% and may be even lower during the first years of diagnosis [35]. A reliable diagnostic test that supports the clinical diagnosis and facilitates the identification of early stages of disease is challenging and unavailable. Numerous efforts have been put into biomarker discovery for PD diagnosis, mostly using CSF due to its potential to reflect changes occurring in the brain [21].

miRNAs are important post-transcriptional regulators of gene expression, with each miRNA predicted to regulate hundreds of target genes and impact multiple cellular processes [36]. miRNAs were first discovered in 1993, and since then their expression pattern has been investigated in different human diseases and recently proposed as diagnostic, prognostic, and treatment response biomarkers [22–25, 37, 38]. In the field of PD, only a handful of studies have proposed miRNAs as potential biomarkers for PD, mostly investigating miRNA expression in the CSF of late stage PD patients [39–41]. Burgos et al. and Gui at al. provided a comprehensive examination of miRNAs and exosomal miRNAs detected in the CSF of late PD patients [39, 40]. However, when considering biomarker discovery with the aim of identifying novel diagnostic tools, both studies present limitations. For example, Gui et al., reported potentially misleading expression results due to the use of small nucleolar RNAs to normalize and quantify miRNAs detected in the CSF [40]. Although Burgos et al. overcame these limitations by using untargeted miRNAs analysis and global signal normalization, the authors focused their analysis particularly on differentially expressed miRNAs in late stage PD compared with controls while not exploring the applicability of miRNAs as biomarkers with diagnostic potential [39].

The availability of literature focusing on the integration of untargeted miRNA profiling, protein expression levels, clinical endpoints and advanced data analysis tools, such as machine learning, in the early stages of PD onset is thus still scarce.

The goal of our study was to explore the potential use of miRNAs as diagnostic tools in the early stages of PD, specifically up to 3 years after initial clinical diagnosis. For this, we developed our methodology based on previous studies published by Burgos et al., and Gui et al., [39, 40].

Through the combination of an optimized exosomal miRNA isolation with small RNA sequencing, We were able to detect 1683 exosomal miRNAs present in CSF. To improve the robustness of our models, we first filtered and excluded all miRNAs with less than 5 read counts, reducing our data set to 389 miRNAs. Subsequently, we excluded miRNAs not expressed in all samples, finalizing with 301 miRNAs. Of these, 121 miRNAs were taken forward for analysis based on their expression pattern and their ability to differentiate controls from early stage PD.

Next, we employed BOSS (Biomarker Optimization Software System), an advanced machine learning platform for discovery and selection of biomarker panels, to identify and group together all miRNAs able to accurately distinguish controls from early stage PD patients. To select the best performing models, we focused on two characteristics: robustness and interpretability. We searched for models that provide a reliable binary diagnosis, control or early stage PD patient, and simultaneously provide insights on how the combination of variables discriminates control from early stage PD. To this end, we combined Fuzzy CoCo with Pareto analysis [33, 42]. Fuzzy CoCo has shown excellent results by dealing with the complexity of biological data while producing small (in terms of manageable number of biomarkers), multivariate, accurate, and interpretable models and has been applied for breast cancer diagnostic [33]. By using Fuzzy CoCo, we filtered and excluded all models with low performance, large number of variables and uninterpretable contextualization. Subsequently, we used Pareto analysis to select the best models based on robustness [42]. Through this advanced machine learning approach, we restricted our analysis from an initial 3200 models to 5 potential biomarker panels.

The selected miRNA biomarker panel, Model A, comprises of Let-7f-5p, miR-27a-3p, miR-125a-5p, miR-151a-3p and miR-423-5p, and the consensus is that early stage PD patients should have high expression levels of Let-7f-5p and low expression levels of miR-27a-3p and miR-423-5p, whereas controls should have high expression levels of miR-125a-5p and low expression levels of miR-151a-3p in the CSF. To the best of our knowledge, none of these miRNAs have been previously proposed as potential biomarkers for PD, but 3 miRNAs are from conserved miRNAs families (Let-7, miR-151 and miR-125), of which these families were reported in either blood or CSF samples from PD patients [40, 43, 44]. When considering neurodegenerative diseases, miR-27a-3p was reported down regulated in Alzheimer’s disease (AD) patients with dementia [45]. To further contextualize our findings in relation to PD pathology, we explored the biological relevance of the miRNAs that make up Model A. We identified 31 pathways involved in PD pathogenesis being regulated by the miRNAs proposed in model A (Figure 3A). Interestingly, the analysis highlighted some regulated pathways previously associated with PD pathogenesis [26–31], suggesting that the miRNAs present in Model A comprise a molecular signature involved in several biological pathways associated with the development of PD.

DJ-1, UCHL1 and α-syn are among the most studied proteins in PD and have been explored as potential biomarkers to differentiate PD from controls [11–14, 46, 47]. The current consensus is that α-syn and UCHL1 concentrations are generally lower in the CSF of late stage PD patients, whereas DJ-1 concentration is higher [11–14, 46, 47]. Our initial analysis revealed that when combining miRNA profiles to DJ-1, UCHL1 and α-syn protein levels, we were able to increase the robustness of the models generated using the approach described above. We initially started our analysis with 1600 models and through an advanced machine learning approach we identified 3 models with high predictive values. From those, Model F was selected based on robustness. Model F is composed of miR-10b-5p, miR-151a-3p, miR-22-3p and α-syn. The interpretation of the model revealed that early stage PD patients should have low α-syn protein levels and, low miR-22-3p expression levels in the CSF, and high expression levels of miR-10b-5p and miR-151a-3p. α-syn and miR-22-3p were previously reported as exhibiting low expression levels in the CSF of PD patients [13, 14, 40]. The pathway analysis of Model F revealed an enrichment of Prion disease pathways (*p* < 0,001), suggesting that this molecular signature has a strong impact in such pathway compared to others (Figure 3B).

After comparing both models, we observed that only one miRNA overlaps between them: miRNA-151a-3p. One potential reason for the differences in the models could be due to α-syn being proposed as bait to miRNAs associated with protein aggregation which could play an important role in introducing changes to the molecular signatures that were identified. This is further elucidated by the fact that miR-10b-5p and miR-22-3p being proposed as regulators of several genes involved with protein aggregation and are predicted to interact with *SNCA*, the α-syn gene [48]. It is also relevant to highlight the challenges around protein analysis, namely when considering α-syn. In our study, we analyzed total α-syn in the CSF and found it expressed in lower levels in the CSF of early stage PD patients compared to controls. Although the majority of publications support this finding, there is still conflicting data available [15]. Furthermore, different isoforms of α-syn have been investigated and proposed as potential biomarkers, including monomeric and phosphorylated forms, among others [9, 49]. As data across different studies is conflicting, it is still unclear which isoform of α-syn could be the most robust endpoint to differentiate controls from PD patients at early or late stages.

Although our findings are promising, further validation in heterogeneous, thoroughly characterized and larger scale cross-sectional studies are needed to further evaluate the robustness of the proposed molecular signatures in the context of early stage PD diagnosis.

## MATERIALS AND METHODS

### Sample collection and patients

Early stage PD patients and controls were recruited from the outpatient clinic at the Neurodegenerative Department of the University of Tübingen, Germany, and clinical data is collated (Table 1). The study was approved by the Ethics Committee of the Medical Faculty of the University of Tübingen (480/2015BO2). All participants provided written informed consent. PD was diagnosed according to the United Kingdom Brain Bank Society Criteria [50]. All patients were investigated by movement disorders specialists, to keep the risk of misdiagnosis at a minimum. Control individuals were assessed as having no neurological disease. Early stage PD patients were chosen to represent a homogeneous cohort with very early disease state (mean disease duration = 2 years, median Hoehn and Yahr stage (H&Y) = 2, and median Unified Parkinson’s disease rating scale III (UPDRS III 6, 29) = 21) and to have the akinetic-rigid subtype of PD [6, 51]. We included only akinetic-rigid patients as there is increasing evidence that tremor-dominant and akinetic-rigid subtypes are the consequence of different pathophysiologies, to increase the probability to find (subtype-) specific results [52, 53]. CSF was collected by lumbar puncture according to standardized guidelines previously described in the literature [54]. To prevent blood contamination, CSF samples were tested for hemoglobin. CSF samples free of blood were centrifuged (1600 g, 4°C, 15 min), frozen within 30–40 min after the puncture and stored at -80°C according to CSF collection and storage guidelines [55].

### RNA extraction

Exosomal RNA was isolated from 250 ul of CSF using miRCURY™ Exosome Isolation Kit serum/plasma kit and miRCURY™ RNA Isolation Kit – Biofluids (Exiqon, Denmark). RNA extraction protocol was optimized to maximize small RNA yield from low input of CSF. RNA was concentrated in 7 µl of RNAse-free water. 2 µl of RNA was used for quality control and concentration assessment using Nanodrop UV-VIS Spectrophotometer (Thermo Fisher Scientific, USA) and Bioanalyzer Small RNA Analysis Kit (Agilent, USA).

### Library preparation and small RNA sequencing

Libraries for small RNA sequencing were prepared using NEB Next small RNA library prep kit (New England Biolabs, USA) following the manufacturer’s instructions with few adjustments to achieve successful sequencing runs from low input RNA. Briefly, 5 µl of RNA was used as input for RNA adapter ligation (using 3′ and 5′ RNA adapters) followed by reverse transcription and PCR amplification (15 cycles) with bar-coded primers. PCR products were pooled based on equal volume prior to size selection on a Pippin Prep system (Sage Science, USA) to recover the 147 nt and 157 nt fractions containing mature miRNAs. The resulting small RNA libraries were concentrated via ethanol precipitation and quantified using the Qubit 2.0 Fluorometer prior to sequencing with read length of 75 bp on a NextSeq 500 sequencer (Illumina, USA). A quality control assessment was performed, using Bowtie [56]. Raw sequencing data was transformed to FastQ format.

### Sequencing processing and normalization

Reads were mapped to the human reference genome (hg38 – UCSC) [57] using Bowtie54. Samples with less than 100,000 mapped reads were removed. Following, mapped reads were assigned to mature miRNAs using genome annotation data from Ensembl (v84) [58], UCSC (hg38) [57] and miRBase (v21) [59]. miRNAs with less than 4 mapped reads, in average, were not considered for further analysis. Raw counts uncertainty was estimated as the 95% tile of the coefficient of variation (CV) per unit of log2-transformed raw counts: for miRNAs with > 64 read counts, the 95% tile CV is < 0.1; for miRNAs with > 32 read counts, the 95% tile CV is < 0.2; and for miRNAs with > 8 read counts, the 95% tile CV is < 0.5. miRNA expression data were normalized using DEseq2 [60].

### Ligand binding assay measurement

Quantitative determination of selected markers was done by ELISA following manufacturer’s guidelines and validated fit-for-purpose as proposed by Jani et al [60]. Total α-syn was measured using mono-kit human α-syn (Analytik-Jena, Germany). 100 µl of CSF were diluted 1:1 in phosphate buffered saline (PBS) pH 7.7 containing 0.05% Tween 20, 3% bovine serum albumin (BSA), 5 mM EDTA and 10 mM PefaBlock. The limit of detection was 0.37pg/mL and the intra-assay precision < 15% CV. DJ-1 was measured using Human DJ-1/PARK7 kit from Meso Scale Discovery (MSD, USA). For DJ-1 measurement, CSF samples were diluted 8-fold; the limit of detection was 12.0 pg/mL and the intra-assay precision was < 10% CV. UCHL1 was measured using Human Neurological Disorders Magnetic Bead Panel 1 from Millipore (Millipore, USA). 25 µl of CSF was used for this assay; the limit of detection was 0.31 ng/mL and the intra-assay precision was < 10% CV.

### Biomarker panel identification

Biomarker panel identification relied on BOSS (Biomarker Optimization Software System), an advanced machine learning platform for discovery and selection of biomarker panels.

Initial pre-processing of the biomarker data included removal of near-zero variance predictors and exclusion of miRNAs expressed in less than 75% of the cohort. The final step before analysis was to randomize subjects into training and test: training (63.5%) and test (36.5%).

BOSS uses a combination of different multivariate methods to build high predictive models of disease status (PD vs. control). Fuzzy modeling and Pareto efficiency were employed to manipulate information in a way that resembles human communication and reasoning processes. Repeated 10-fold cross validation of the training set was used to give an indication of the accuracy of the resulting predictive models. The models were then applied to the data in the test set and predictive probabilities were generated. Confusion matrices were produced and model fit was assessed using the following parameters: sensitivity, specificity and area under the Receiver Operating Characteristics (ROC) curve.

### Analysis of target genes

DIANA-mirPath was used to perform target prediction and pathway analysis based on miRTarBase [34, 61]. The software performs an enrichment analysis of multiple miRNA target genes to Kyoto Encyclopedia of Genes and Genomes (KEGG) pathways. The statistical significance value associated with the identified biological pathways was calculated by mirPath [34].

### Statistics

Demographic and baseline characteristics of the cohorts were assessed using summary statistics. Differences in means between early stage PD and Controls were assessed using *T*-test; differences in proportions were assessed using chi-squared tests.

## CONCLUSIONS

In this study, we demonstrated that miRNAs are detectable in abundance in CSF exosomes and demonstrate the importance of dedicated data analysis to explore their potential as reliable diagnostic biomarkers to be deployed at the early stages of PD. To the best of our knowledge, this is the first study to integrate state-of-the-art microRNA sequencing with protein analysis and complex machine learning. We propose two robust biomarker panels that efficiently distinguish early stage PD patients from controls. In addition, we showed that both panels are characterized by regulators of the key mechanisms of PD pathology.

## SUPPLEMENTARY MATERIALS FIGURE AND TABLES


