# Supplementary material for: miRNA-based signatures in cerebrospinal fluid as potential diagnostic tools for early stage Parkinson’s disease
Source: Oncotarget. 2018 Apr 3;9(25):17455–65. doi: 10.18632/oncotarget.24736 (PMC5915128; doi:10.18632/oncotarget.24736)
Supplement: Supplementary file 1 [file oncotarget-09-17455-s001.pdf]

miRNA-based signatures in cerebrospinal fluid as potential diagnostic tools for early stage Parkinson's disease

SUPPLEMENTARY MATERIALS

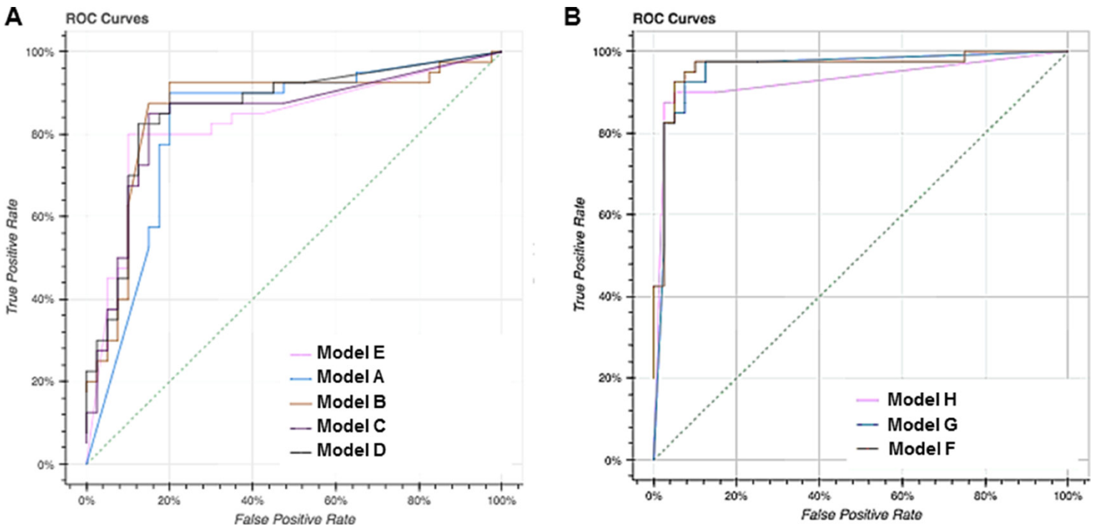

Supplementary Figure 1: Diagnostic accuracy of potential models. (**A**) ROC curve of miRNA-based models with high sensitivity and specificity values - selected Model A (blue line) and supplementary Models B (brown line), C (purple line), D (black line) and E (pink line). (**B**) ROC curves of selected miRNA+α-syn Model F (brown line) and supplementary Models G (blue line) and H (pink line).

Supplementary Table 1: Predictive values of miRNA-based models with sensitivity and specificity values above 80%

Model	SEN	SPE	AUC	PPV	NPV
A	90.00%	80.00%	82.31%	81.82%	88.89%
B	87.50%	85.00%	85.88%	85.37%	87.18%
C	85.00%	85.00%	84.22%	85.00%	85.00%
D	82.50%	87.50%	86.03%	86.84%	83.33%
E	80.00%	90.00%	82.94%	88.89%	81.82%

Sensitivity (SEN), Specificity (SPE), Area under the curve (AUC), Positive predictive value (PPV) and Negative Predictive Value (NPV).

Supplementary Table 2: Predictive values of models including miRNAs and α-syn with sensitivity and specificity values above 90%

Model	SEN	SPE	AUC	PPV	NPV
F	97.50%	90.00%	96.19%	90.70%	97.30%
G	92.50%	92.50%	95.72%	92.50%	92.50%
H	90.00%	95.00%	92.94%	94.74%	90.48%

Sensitivity (SEN), Specificity (SPE), Area under the curve (AUC), Positive predictive value (PPV) and Negative Predictive Value (NPV).
